# Airway Smooth Muscle Hypercontractility in Asthma

**DOI:** 10.1155/2013/185971

**Published:** 2013-03-18

**Authors:** Rachid Berair, Fay Hollins, Christopher Brightling

**Affiliations:** Department of Infection, Inflammation and Immunity, Institute for Lung Health, University of Leicester, Leicester LE3 9QP, UK

## Abstract

In recent years, asthma has been defined primarily as an inflammatory disorder with emphasis on inflammation being the principle underlying pathophysiological characteristic driving airway obstruction and remodelling. Morphological abnormalities of asthmatic airway smooth muscle (ASM), the primary structure responsible for airway obstruction seen in asthma, have long been described, but surprisingly, until recently, relatively small number of studies investigated whether asthmatic ASM was also fundamentally different in its functional properties. Evidence from recent studies done on single ASM cells and on ASM-impregnated gel cultures have shown that asthmatic ASM is intrinsically hypercontractile. Several elements of the ASM contraction apparatus in asthmatics and in animal models of asthma have been found to be different from nonasthmatics. These differences include some regulatory contractile proteins and also some components of both the calcium-dependent and calcium-independent contraction signalling pathways. Furthermore, oxidative stress was also found to be heightened in asthmatic ASM and contributes to hypercontractility. Understanding the abnormalities and mechanisms driving asthmatic ASM hypercontractility provides a great potential for the development of new targeted drugs, other than the conventional current anti-inflammatory and bronchodilator therapies, to address the desperate unmet need especially in patients with severe and persistent asthma.

## 1. Introduction

Asthma is a chronic inflammatory disease characterized by variable airflow obstruction and bronchial hyperreactivity associated with airway remodelling [1]. Most of asthma symptoms result from airflow obstruction caused by airway lumen narrowing. Although this narrowing is multifactorial in origin, abnormalities of airway smooth muscle (ASM) structure and function have been identified as one of the main causes [2]. Increased ASM mass has long been recognized as a major component of airway remodelling [3, 4]. More recently, asthmatic ASM was also found to be abnormal in its functional properties with increasing evidence showing intrinsic heightened contractility independent of other structural cells and independent of the asthma inflammatory milieu. In this paper we will examine the evidence of ASM hypercontractility in asthmatics, explore the potential mechanisms driving it, discuss its relevance, and briefly suggest its role in future asthma therapy.

## 2. Evidence of ASM Hypercontractility in Asthmatics

Abnormalities of asthmatic ASM structure and morphology have been described by Huber and Koesser more than 90 years ago when they reported increased ASM mass in a small group of patients who died of status asthmaticus compared to ASM form patients who died from nonpulmonary conditions [3]. This structural association has since been extensively described [4] although whether asthmatic ASM is also abnormal in function and if so whether this abnormality is an inherent property or only a result of the asthma inflammatory milieu has long been an unresolved question [5]. A few *in vitro* studies from the 1980–90s have tried to address this issue but the results have largely been conflicting. Compared to nonasthmatic controls, some studies suggested increased force generation in asthmatics ASM preparations; others showed no difference and even some seem to suggest decreased force generation in asthmatics [6–13]. Most of these studies had major methodological and statistical limitations such as small sample size, failure to measure force per cross-sectional area (stress), failure to measure ASM shortening, and failure to identify ideal lengths for maximal contraction [14]. Furthermore, none of these studies examined ASM contractility at a cellular level, thus the mechanical effect of the extracellular nonmuscular connective tissue, and the biological effect of inflammatory cells and cytokines present in ASM preparations, on the final results could not be determined.

The first robust evidence of ASM hypercontractility in asthmatics was reported by Ma et al. This was the first study attempting to assess contractility characteristics of asthmatic ASM at cell level [15]. The study included 5 asthmatics and a similar number of nonasthmatic controls. 10–20 ASM cells were isolated from endobronchial biopsies collected from each subject. Maximum capacity and velocity of shortening of zero loaded single ASM cells in response to electrical field stimulation were measured under inverted phase-contrast microscopy. Asthmatics ASM cells showed significantly increased maximum capacity and velocity of shortening compared to controls. Although in this study the maximum shortening capacity in asthmatic ASM cells was increased by almost a third compared to controls, it should be considered that this shortening was measured at zero load and ASM cells *in vivo* would very likely shorten by a much lesser degree. This observation is pivotal but needs to be interpreted with caution due to the small sample size of this study.

Matsumoto et al. assessed asthmatic ASM contractility using a collagen gel contraction assay [16]. Gel percentage contraction to histamine was measured using floating gels containing ASM from 8 subjects with asthma and 9 nonasthmatic controls. These ASM containing gels were incubated overnight using 2 methods: attached or unattached to casting plates. The study found, using both methods, that histamine-exposed gels containing asthmatic ASM contracted more significantly.

**Figure 1:**
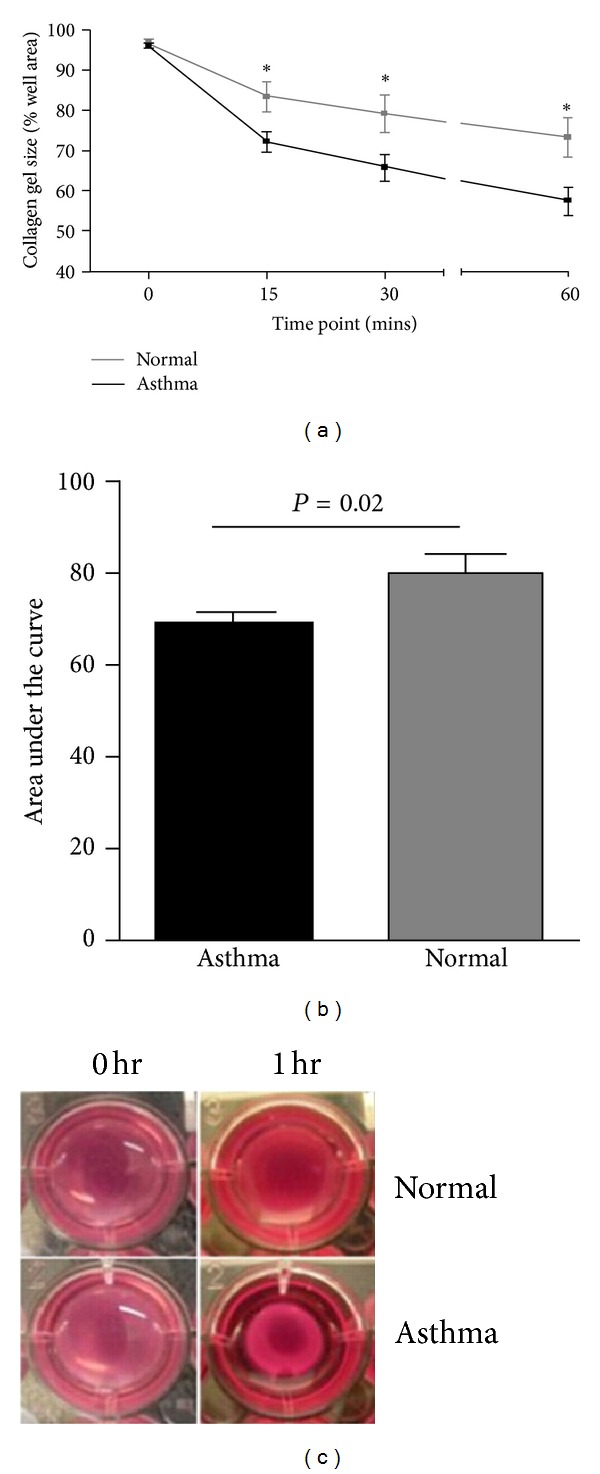
(a) Percentage contraction of collagen gels impregnated with airway smooth muscle from donors with asthma (*n* = 19) versus healthy control donors (*n* = 8) over 1 hour following stimulation with 1 nm bradykinin, (b) area under the curve gel contraction (mean [SEM]), and (c) representative gel photographs taken at 0 hour and 1 hour time points. The comparison was made by unpaired *t-*test. *P < 0.05. [17]. (Reprinted with permission of the American Thoracic Society. Copyright © 2012 American Thoracic Society. Amanda Sutcliffe, Fay Hollins, Edith Gomez, Ruth Saunders, Camille Doe, Marcus Cooke, R. A. John Challiss, and Chris E. Brightling/2012/ Increased Nicotinamide Adenine Dinucleotide Phosphate Oxidase 4 Expression Mediates Intrinsic Airway Smooth Muscle Hypercontractility in Asthma. American Journal of Respiratory and Critical Care Medicine/Vol. 185/pp 267-274. (An official Journal of The American Thoracic Society).

More recently, Sutcliffe et al. also used gel contraction assay to assess ASM contractility in a much larger sample of 19 asthmatics and 8 healthy controls [17]. Gel contraction was measured every 15 minutes after stimulation with bradykinin. Results again showed significantly increased agonist-induced contraction in the asthma group (Figure 1).

Importantly, phenotypic plasticity of structural cells in culture cannot be completely excluded from studies done on primary ASM cultures, but we think this, if present, was minimal. The above evidence, in our opinion, confirms that asthmatic ASM is fundamentally different and hypercontractile and that this hypercontractility is a basic property and is independent from other asthma structural cells and airway inflammation, although *in vivo* these may play an important role in modulating the hypercontractile response.

## 3. Potential Mechanisms Driving ASM Hypercontractility in Asthmatics

### 3.1. Physiology of Human ASM Contraction

As in all muscle cells, contraction in ASM is initiated by increased cytosolic calcium ions (Ca^+2^) level, though, unlike most other muscle cells, the source of this Ca^2+^  surge in ASM is mainly from intracellular sarcoplasmic reticulum (SR) stores rather than from the usual extracellular Ca^2+^ influx through voltage-dependent calcium channels during depolarization seen in cardiac, skeletal, and vascular muscle cells.

**Figure 2:**
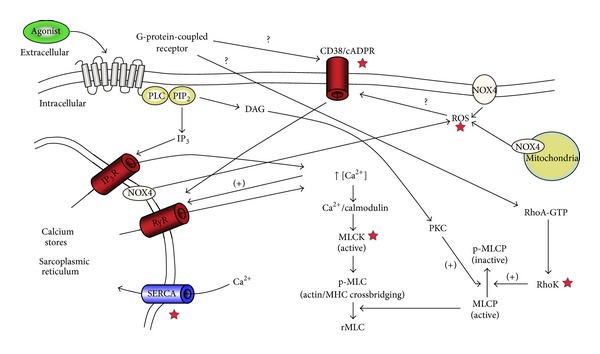
Overview of the signalling pathways involved in airway smooth muscle contraction. The contractile agonist interacts with its specific G-protein-coupled receptor (GPCR) leading to the activation of phospholipase C (PLC) which hydrolyzes phosphatidylinositol bisphosphate (PIP_2_) leading to the formation of two-second messengers, inositol triphosphate (IP_3_), and diacylglycerol (DAG). IP_3_ interacts with its receptor (IP_3_R) on the sarcoplasmic reticulum (SR) leading to the release of calcium ions Ca^2+^ which in turn, through forming a complex with calmodulin, activates myosin light chain kinase (MLCK). MLCK phosphorylates regulatory myosin light chain (rMLC) to form p-MLC which leads to myosin and actin crossbridging and contraction. p-MLC is deactivated by the action of myosin light chain phosphatase (MLCP). Both DAG, through its action on protein kinase C (PKC), and RhoA, through its target Rho Kinase (RhoK), have an inhibitory action on MLCP through phosphorylation. Interaction of agonist with GPCR also activates both CD38/cADPR and Rho/RhoK pathways, although the exact mechanism is not fully known. CD38/cADPR activation leads to the release of Ca^2+^ from SR through ryanodine receptors (RyR) channels. Nicotinamide adenine dinucleotide phosphate oxidase type 4 (NOX4) generates reactive oxygen species (ROS) which may affect ASM calcium homeostasis and subsequently ASM contraction through their action on the CD38/cADPR pathway. Red stars ★ indicate signalling points with abnormalities suspected of driving hypercontractility in asthmatic ASM.

The sequence of events leading to the contraction of an ASM cell starts with the interaction of a contractile agonist with its G-protein-coupled receptors (Figure 2). This results in the activation of phospholipase C (PLC), which in turn leads to the formation of the inositol triphosphate (IP_3_) through the hydrolyzation of phosphatidylinositol bisphosphate (PIP_2_). IP_3_ then binds to its receptor on SR membrane releasing Ca^2+^ stores which then, through forming a complex with calmodulin, activate myosin light chain kinase (MLCK) which phosphorylates regulatory myosin light chains (rMLC) forming p-MLC [18]. Finally, this leads to the activation of actin and myosin crossbridges resulting in shortening and contraction [19]. After initiation of contraction, cytosolic Ca^2+^ levels return to normal through different mechanisms including pumping out of the cell by the plasma membrane Ca^2+^-ATPase (PMCA) and the sodium calcium exchanger (NCX), binding to cytosolic proteins, uptake by mitochondria, and also reuptake to the SR through the action of the sarco/endoplasmic reticulum Ca^2+^ ATPase (SERCA) [20].

Another mechanism for agonist-induced Ca^2+^ released from SR is through ryanodine receptors (RyR). This is mediated by membrane CD38 and nucleotide metabolite cyclic ADP-ribose (cADPR) [21]. The RyR channels are also activated through localized elevation of Ca^2+^ levels (Ca^2+^ induced Ca^2+^ release).

The phosphorylation of rMLC is also regulated by myosin light chain phosphatase (MLCP) which converts p-MLC back to inactive rMLC. MLCP activity is modulated through two agonist-induced mechanisms in a process called calcium sensitization. First, it is controlled by the inhibitory action of diacylglycerol (DAG), another second messenger, which also results from the hydrolyzation of PIP_2_. DAG activates Protein Kinase C (PKC) which in turn inhibits MLCP through phosphorylation. Second, MLCP is also negatively controlled by RhoA and its target Rho Kinase, which deactivates MLCP similarly through phosphorylation.

Exploring the possible mechanisms of asthmatic ASM hypercontractility is a difficult task as the evidence is less well established with relatively few human studies. Hypercontraction of asthmatic ASM could be due to abnormalities in one or more of these components or steps of ASM contraction model. The complexity of investigating differences in signalling or contractile proteins is that the abnormality could be in a number of levels. This could be at gene, gene expression (epigenetics), or, more commonly, at protein phosphorylation level.

### 3.2. Abnormalities of Contractile Proteins

The most characterized potentially abnormal component of the contraction apparatus in asthmatic ASM is MLCK, a key regulator of ASM contraction. Increased MLCK levels have been reported in sensitized animal and human airways [22, 23]. As part of the same contractility study described earlier, Ma et al. assessed MLCK expression by using RT-PCR to measure mRNA. Measuring MLCK protein was not possible due to the small cell sample (10–20 cells per subject) [15]. MLCK mRNA was significantly increased in asthmatics compared to a group of both allergic and nonallergic nonasthmatic controls.

Benayoun et al. examined contractile protein expression in biopsies in asthmatics with different asthma severity compared to nonasthmatic controls and also compared to patients with COPD [24]. Although *α*-actin and myosin heavy chain isoforms (SM1, SM2) expression was similar in all groups, MLCK expression was increased in all patients with asthma and COPD compared to controls. Furthermore, MLCK expression was significantly more in patients with severe asthma compared to all other groups. Interestingly, although p-MLC, the active product from the action of MLCK, was detected only in the asthmatic groups, this was not statistically significant.

Not all studies showed increased MLCK expression or content in asthmatics. Matsumoto et al. demonstrated no increase in MLCK content in cultured asthmatic ASM. The authors did admit that this negative result could be due to possible degradation of MLCK during the harvesting stage of ASM cells [16]. Moreover, Woodruff et al. showed no increased gene expression of any of the contractile proteins MLCK, MCH, SM22, or *α*-actin in a sample of 11 asthmatics compared to 8 controls, although this could be due to the fact that the asthmatics in this study had only mild disease [25].

*In vivo*, the degree of mast cell infiltration of ASM, a histopathological feature of asthma [26, 27], has been shown to be positively associated with increased *α*-actin expression [28]. Moreover, *in vitro* coculture of human ASM with human lung mast cell (HLMC), or *β*-tryptase, a serine protease released by mast cells following activation, resulted in increased *α*-actin expression and increased ASM contraction. This has been shown to be mediated through autocrine upregulation of transforming growth factor *β*1 (TGF-*β*1) in ASM [28]. Histamine release from mast cells in a piece-meal fashion as demonstrated by mast cells within the ASM bundle *in vivo* by electron microscopy [27] and *in vitro* following fibroblastoid differentiation [29, 30] might exert a direct spasmogen effect upon the ASM in asthma. Mast cells are also an important source of cytokines including the Th2 cytokine IL-13 [31]. Mast cells localized in the ASMbundle express IL-13, particularly in severe disease which can prime ASM to a more hyper-contractile state [31–33]. Abnormalities of *α*-actin expression in asthmatic ASM have only been described in this context and previous studies examining *α*-actin expression in asthmatics were mostly negative [24, 34].

### 3.3. Dysregulation of Calcium Homeostasis

As discussed earlier, calcium plays a central role in ASM contraction. There is an increasing pool of evidence showing abnormal calcium homeostasis in asthmatic ASM, thus, suggesting that abnormal calcium handling, signaling, or storage as possible underlying mechanisms for asthmatic ASM hypercontractility is a plausible argument. 

In general, factors leading to increased cytosolic Ca^2+^ levels result in increased ASM contraction. Mahn et al. examined SERCA expression in ASM from asthmatics and healthy volunteers [35]. The expression of SERCA2 isoform mRNA, the main isoform expressed in human ASM, was reduced in patients with moderately severe asthma. This was found in *in vivo* samples and also on ASM cultures. Reduction of SERCA2 expression in healthy control ASM culture by siRNA resulted in phenotypic shifting to an asthmatic ASM type with increased motility, secretion, and slow Ca^2+^ recovery. Of interest, IP_3_R mRNA expression was not increased in the asthmatics group.

Another signalling pathway that has the potential of altering calcium homeostasis and increasing contractility is the CD38/cADPR/RyR pathway. CD38 deficiency in animals has been shown to inhibit airway hyperresponsiveness (AHR) [36]. In human ASM, TNF-*α*, IL-1*β*, IL-13, and IFN-*γ* were all found to increase CD38 expression, cADPR activity, and Ca^2+^ response to various natural contractile agonists [37, 38]. Moreover, in another study, highlighting the possibility that CD38 abnormalities could be a fundamental characteristic in asthma, TNF-*α* was shown to significantly increase CD38 expression in asthmatic ASM than in controls [39].

Altered calcium homeostasis in asthmatic ASM is also due to altered extracellular calcium influx through non-voltage-dependent channels [40]. Although this has been directly implicated in altered mitochondrial biogenesis and increased ASM proliferation in asthmatics, its relevance to hypercontractility remains to be investigated.

### 3.4. Abnormal Calcium Sensitization

Upregulation of the calcium independent RhoA/Rho Kinase signalling pathway leading to inhibition of MLCP would result in increased levels of p-MLC and subsequently increased ASM contraction force at the same Ca^2+^ concentration. Abnormalities of this signalling pathway have been described in animal models of various smooth muscle disorders including hypertension, coronary artery spasm, and preterm labour [41]. This has also been described in animal models of allergic bronchial asthma [42]. Increased levels of RhoA protein and RhoA mRNA were found in airway hyperresponsive rat models although this is probably medicated through inflammatory cytokines [41–43].

We emphasize that most of the abnormalities of calcium homeostasis and calcium sensitization explored in this paper were only described in single studies which have not been replicated; thus their importance remains to be fully determined.

### 3.5. Increased Oxidative Stress Burden

Although reactive oxygen species (ROS) play an important physiological role in different cellular functions, excessive production results in the tissue damage seen in a range of chronic and acute diseases. Oxidative stress burden is increased in bronchial asthma with recent evidence identifying an increase in the generation of ROS in asthmatic ASM *in vivo* and in primary ASM cultures [17]. This was inversely correlated to the degree of airflow obstruction and AHR. More importantly, nicotinamide adenine dinucleotide phosphate oxidase type 4 (NOX4) expression, an important source of ROS, was increased in asthmatics ASM. Moreover, increased asthmatics ASM contractility seen in gel contraction essay was abolished by adding NOX4 inhibiters or NOX4 small interfering RNA. 

### 3.6. SMAD3 and ORMDL3

Genome-wide association studies (GWASs) have identified several associations between multiple single-nucleotide polymorphisms (SNPs) on a number of locations and asthma. One such SNP is on the *SMAD3 gene*. *SMAD3 gene, *located on chromosome 15, encodes for a similarly named protein, SMAD3 protein. This protein is a signal transduction and transcription modulator and is activated by TGF-*β* which has a complex role in cell growth and proliferation and is involved in airway inflammation and remodelling in asthma. As mentioned earlier, upregulation of TGF-*β*1 observed on coculture of ASM and HLMC was associated with increased *α*-actin expression and increased contractility of ASM [28]. 

Another association identified by GWASs was on the *ORMDL3 gene *[44, 45]. This gene encodes for the SR membrane protein ORMDL3 which is thought to have an important role in calcium homeostasis possibly through its action on SERCA. Overexpression of ORMDL3 was found to be associated with reduced SERCA activity as evidenced by higher basal cytosolic Ca^2+^ levels, lower SR Ca^2+^ levels, and slower Ca^2+^ reuptake into the SR [46]. Thus, based on the above evidence, polymorphism of *SMAD3* or *ORMDL3 *could be implicated in the hypercontractility seen in asthmatic ASM through their effect on *α*-actin expression and calcium homeostasis, respectively.

## 4. Clinical Relevance of Asthmatic ASM Hypercontraction

The contraction of ASM in asthma causes airway obstruction [47]. Although we know that AHR is predominantly a function of ASM, how much of it is driven by ASM hypercontractility is a difficult and controversial question to answer. We do recognize that some of ASM hypercontractility is contributed to by airway inflammation and inflammatory cytokines, but there is also good evidence to suggest that there is more to AHR than inflammation. Although corticosteroids reduce AHR, studies using agents that target specific parts of inflammation, in the form of antibodies against IL-5 and IgE, significantly improved inflammation but did not affect AHR [48–50].

Therapies that reduce ASM contraction have been shown to improve asthma symptoms, lung function, and maybe even AHR. The roles of *β*2-agonists and anticholinergics in the treatment of asthma are well established. Another evidence of the significance of ASM is from bronchial thermoplasty (BT). BT, where radiofrequency energy is used on airways to reduce ASM mass, was shown to improve asthma control, quality of life, and, in one study, AHR [51]. Further studies are required to examine whether the efficacy of BT in asthmatics is related to changes in ASM mass and how this relates to changes in airway structure, physiology, and clinical expression of disease.

## 5. ASM Hypercontractility and the Future of Asthma Therapy

The desperate need for new asthma treatments is universally acknowledged. Asthma incidence is increasing with more than half of the patients failing to achieve adequate control. Furthermore, 5–10% of patients have persistent symptoms despite maximal treatment with conventional anti-inflammatory and bronchodilator therapy [1, 52].

Detailed discussion of the future of asthma treatment is beyond the scope of this paper. Targeted drugs that would act on specific aspects of inflammation and contractility are the way forward. Over the last few years, a huge research effort has been on trying to identify asthma phenotypes based on inflammation, but much less was dedicated to addressing contractility. Several chemicals have been found to reduce ASM gel contraction *in vitro* including inhibitors of phospholipase C, myosin light chain kinase, Rho kinase, and NOX4 and thus this has identified these enzymes as potential targets for future novel asthma treatments [16, 17]. 

## 6. Conclusion

In conclusion, we believe, based on the evidence reviewed, that ASM in asthmatics is hypercontractile. Airway inflammation contributes to and augments ASM hypercontractility but is neither sufficient nor necessary. Indeed evidence presented here suggests that ASM hypercontractility is an intrinsic abnormality in asthma that persists in primary culture in the absence of the asthmatic environment. Improved understanding of the mechanisms driving this hypercontractility will pave the way for future treatments that will address contractility, achieve better relief of airway obstruction, and impact on asthma control and exacerbations.
